# Differential Antibody Response to Inactivated COVID-19 Vaccines in Healthy Subjects

**DOI:** 10.3389/fcimb.2021.791660

**Published:** 2021-12-16

**Authors:** Jiaqi Zhang, Shan Xing, Dan Liang, Wei Hu, Changwen Ke, Jinyong He, Runyu Yuan, Yile Huang, Yizhe Li, Dongdong Liu, Xuedong Zhang, Lin Li, Jianhua Lin, Weili Li, Xiangyun Teng, Yijun Liu, Wei Wen, Qiang Kang, Dawei Wang, Wanli Liu, Jianhua Xu

**Affiliations:** ^1^ Department of Laboratory Medicine, Shunde Hospital of Guangzhou University of Chinese Medicine, Foshan, China; ^2^ Department of Laboratory Medicine, The Second Affiliated Hospital of Guangzhou University of Chinese Medicine, Guangzhou, China; ^3^ Department of Laboratory Medicine, State Key Laboratory of Oncology in South China, Collaborative Innovation Center for Cancer Medicine, Sun Yat-sen University Cancer Center, Guangzhou, China; ^4^ Guangdong Center for Disease Control and Prevention, Guangdong Provincial Institute of Public Health, Guangzhou, China; ^5^ Department of Medical Affairs, Autobio Diagnostics Co. Ltd, Zhengzhou, China; ^6^ Research & Development Centers, Autobio Diagnostics Co. Ltd, Zhengzhou, China; ^7^ Health Management Research Center, Shunde Hospital of Guangzhou University of Chinese Medicine, Foshan, China; ^8^ Emergency Department, Shunde Hospital of Guangzhou University of Chinese Medicine, Foshan, China; ^9^ Department of Pulmonary and Critical Care Medicine, Shunde Hospital of Guangzhou University of Chinese Medicine, Foshan, China

**Keywords:** antibody response, inactivated SARS-CoV-2 vaccine, neutralizing antibody, antibody dynamic, immune response

## Abstract

The appearance and magnitude of the immune response and the related factors correlated with SARS-CoV-2 vaccination need to be defined. Here, we enrolled a prospective cohort of 52 participants who received two doses of inactivated vaccines (BBIBP-CorV). Their serial plasma samples (n = 260) over 2 months were collected at five timepoints. We measured antibody responses (NAb, S-IgG and S-IgM) and routine blood parameter. NAb seroconversion occurred in 90.7% of vaccinated individuals and four typical NAb kinetic curves were observed. All of the participants who seroconverted after the first dose were females and had relatively high prevaccine estradiol levels. Moreover, those without seroconversion tended to have lower lymphocyte counts and higher serum SAA levels than those who experienced seroconversion. The NAb titers in young vaccine recipients had a significantly higher peak than those in elderly recipients. S-IgG and S-IgM dynamics were accompanied by similar trends in NAb. Here, we gained insight into the dynamic changes in NAbs and preliminarily explored the prevaccine blood parameters related to the kinetic subclasses, providing a reference for vaccination strategies.

## Introduction

As of May 29th, 2021, there have been more than 170 million worldwide confirmed cases of coronavirus disease 2019 (COVID-19), which is caused by severe acute respiratory syndrome coronavirus 2 (SARS-CoV-2), and the pandemic has caused more than 5.1 million deaths (https://www.worldometers.info/coronavirus). Vaccines are the most powerful weapon for preventing infectious diseases (Walsh et al., 2020; Zhu et al., 2020; Pan H.-X. et al., 2021; Xia et al., 2021). Immunogenicity and safety assessments postvaccination and monitoring the dynamic human humoral response to SARS-CoV-2 vaccination are important for informing public policy and developing vaccination strategies.

The immunological response to SARS-CoV-2 vaccination is often evaluated by monitoring the presence of total binding antibodies and neutralizing antibodies (NAbs) (De Marinis et al., 2020; Tang M. S. et al., 2020; Elledge et al., 2021; Yao et al., 2021). NAb levels have typically been used as the gold standard for evaluating the efficacy of vaccines, such as those against poliomyelitis, smallpox and influenza viruses (Van Remmerden et al., 2012), and NAbs against SARS-CoV-2 have been considered a good indicator of protective immunity in multiple studies (Zinkernagel, 2003). The immunogenicity of SARS-CoV-2 inactivated vaccines has been evaluated in several studies (Walsh et al., 2020; Zhang et al., 2020; Al Kaabi et al., 2021; Pan et al., 2021a). Nevertheless, the limitations of these studies are the lack of analysis of the individual-specific dynamic changes in NAbs postvaccination and possible related factors.

Sinopharm COVID-19 vaccine (BBIBP-CorV), an inactivated vaccine, developed by the Sinopharm and the Beijing Institute of Biological Products Co. has been granted emergency use by the World Health Organization and administered worldwide. Here, we quantified how the levels of NAbs, SARS-CoV-2 spike-specific IgG and IgM (S-IgG and S-IgM)changed in the months following BBIBP-CorV vaccination by examining longitudinal samples collected prevaccination, at 14 and 28 days after the first dose, at 14 and 28 days after the second dose from a prospective cohort of 52 vaccine recipients. NAb results for these subjects were obtained by micro-neutralization test, a conventional live virus neutralization (cVNT), which is recognized as the gold standard method test (Muruato et al., 2020; Tan et al., 2020). We aimed to explore the kinetics of NAb development and the prevaccine clinical characteristics associated with the immune response. Such detection and analysis of NAb activity following vaccination can, therefore, provide a reference for mass vaccination strategies.

## Methods

### Subjects

**Figure 1:**
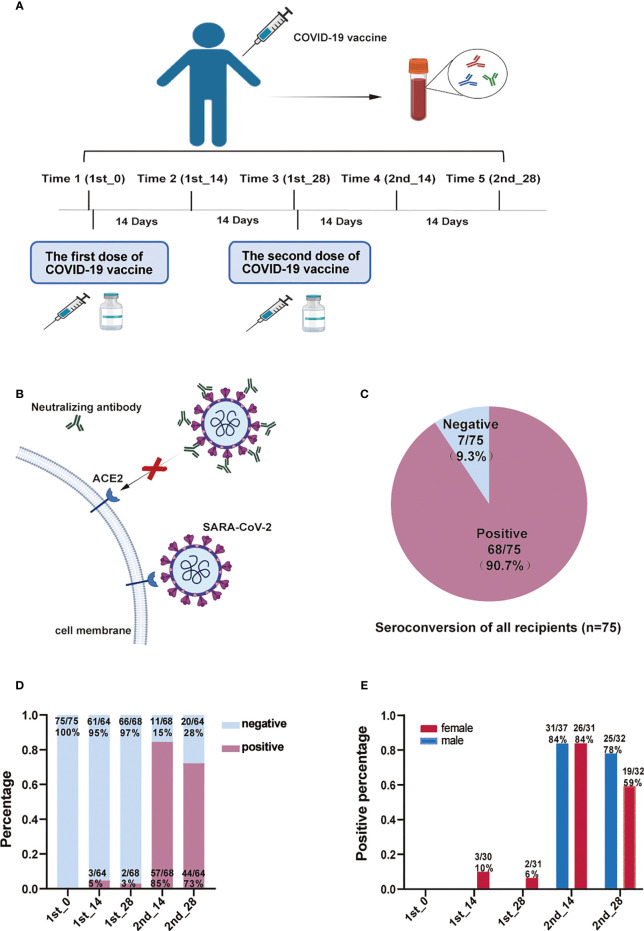
Experimental study scheme and anti-SARS-CoV-2 neutralizing antibody detection. **(A)** The experimental scheme of this study. **(B)** The mechanism of the micro-neutralization test. Anti-SARS-CoV-2 neutralizing antibodies bind to the virus and prevent it from recognizing the ACE2 receptor. **(C)** NAb seroconversion rates of all recipients (n=75). **(D)** The proportion of NAb titer-positive recipients at different time points after vaccination. **(E)** The proportion of NAb titer-positive males and females at different time points after vaccination. COVID-19, Coronavirus Disease 2019; ACE2, angiotensin-converting enzyme 2; NAb, anti-SARS-CoV-2 neutralizing antibody.

To study longitudinal changes in NAb production, venous blood (3-5 ml) was collected from 75 vaccination recipients at five time points: prevaccination, 14 days and 28 days after the first dose, and 14 days and 28 days after the second dose (**Figure 1A**). The blood samples were allowed to clot at room temperature for 30 mins and centrifuged at 1000 x g for 15 min; to avoid repeated freeze-thaw cycles, the serum was aliquoted within 3 h and stored at -80°C until use.

The protocol and informed consent of the study were reviewed and approved by the Medical Ethical Committee of ShunDe Hospital of Guangzhou University of Chinese Medicine (Approval No.: KY2020001/2020128). Before screening for eligibility, written informed consent was obtained from each volunteer. Participants enrolled were healthy adults who were confirmed as SARS-CoV-2 nucleic acid negative by pharyngeal swab reverse transcription polymerase chain reaction.

### COVID-19 Vaccination

Subjects received two intramuscular injections, 28 days apart, delivered in the deltoid muscle. Each injection contained 4 µg/0.5 mL of Sinopharm COVID-19 vaccine (BBIBP-CorV), which is an inactivated SARS-CoV-2 vaccine (Vero cells) from the Sinopharm and the Beijing Institute of Biological Products Co.

### Data Collection and Laboratory Analysis

The sex, age, BMI, adverse reactions and other clinical characteristics of each vaccination recipient were collected. Our study detected SARS-CoV-2 spike-specific IgG and IgM (S-IgG and S-IgM), routine hematologic parameters at each timepoint and measured biochemical markers of the enrolled participants prevaccination. Levels of S-IgG and S-IgM were assessed using a chemiluminescent assay kit according to the manufacturer’s instructions (Autobio Diagnostics, Zhengzhou, China). S/CO values were obtained, and a value ≥ 1.0 was qualitatively defined as positive. Hematologic markers were detected using a Sysmex XN2000 analyzer (Sysmex, Japan). Levels of liver function markers were tested using Roche kits on a Roche Cobas 702 biochemical analyzer (Germany). Serum levels of inflammatory markers, serum amyloid A (SAA) and C-reactive protein (CRP) were measured using latex enhanced immune turbidimetry kits (SAA, Weimi Bio-Tech, China; CRP, SEKISUI, Japan) on an automatic biochemical analyzer (Roche Cobas 702, Germany), while estradiol (E2) and thyroid-related hormones were assessed using Roche kits (Germany) by an electrochemiluminescence analyzer (Roche Cobas 602, Germany). All tests were performed according to the manufacturer’s protocols.

### Micro-Neutralization Test

A micro-neutralization test was conducted at the Centers for Disease Control and Prevention of Guangdong (CDC). The mechanism is shown in **Figure 1B**. First, 2.0 x 10^5^ VERO-E6 cells per well in 100 μL culture media were seeded in 96-well plates. Serum samples after inactivation, four-fold serial dilutions, starting from 1:4 to 1:1024. Then, 125 μL diluted serum was preincubated with the same volume of SARS-CoV-2 suspension of 100 TCID50 per mL for 120 min at 37°C in a 5% CO2 incubator. Then, 100 μL/well virus-serum mixtures were added to monolayer Vero-E6 cells at 37°C in a 5% CO2 incubator. After 4 days of culture, the cytopathic effect (CPE) of each well was recorded under a microscope by two independent observers. The highest dilution that protected more than half of the cells from CPE was regarded as the NAb titer. An NAb titer equal to or above 1:4 was defined as positive (Marklund et al., 2020).

### Statistical Analysis

All statistical analyses were conducted with GraphPad Prism version 8.0 software (GraphPad Software™, San Diego, CA, USA) and SPSS 23.0 (IBM Corporation, Armonk, NY, USA). Seroconversion was defined as a change from seronegative (< 1:4) to seropositive (≥ 1:4). Medians with minimum and maximum are used to describe continuous variables and numbers with percentages for categorical variables. A nonparametric Kruskal-Wallis rank-sum method was employed to compare differences in multiple groups, and a nonparametric Mann-Whitney U test was applied to analyze differences between the two groups. Comparison of categorical data was performed using the chi-squared test or Fisher’s exact test. Correlations between NAb titers and age or NAb titers with other parameters, including S-IgG and S-IgM, were evaluated by Pearson’s correlation coefficient. All tests were two-sided, and a *P* value less than 0.05 was considered statistically significant.

## Results

### Healthy Subjects That Received COVID-19 Inactivated Vaccine

We recruited 75 subjects who received two injections of BBIBP-CorV. To explore the dynamic changes in NAbs, we collected longitudinal serum samples at 5 timepoints (**Figure 1A**), as described in the methods. Subjects were tested with a traditional live virus-based neutralization assay (**Figure 1B**). NAbs were negative at the prevaccine baseline, and 90.7% of the vaccine recipients seroconverted after the administration of the two doses of the vaccine (**Figure 1C**). The proportion of samples with a Nab titer equal to or greater than 1:4 increased slightly with time, peaking at day 14 after the second dose and decreasing slightly thereafter (**Figure 1D**). Moreover, positive results for NAb were detected in more female subjects than males after the first dose, indicating that the females yielded NAbs earlier than the males after vaccination (**Figure 1E**). However, after the second dose, the positive rate of NAb was comparable in both groups.

Samples were collected at all timepoints for 52 subjects, whereas the remaining 23 participants were lost to follow-up at some timepoints and thus excluded from the following analysis. Demographic and health condition data for the vaccine recipients are summarized in **Table 1**. This cohort included 28 (53.8%) males and 24 (46.2%) females, with a median age of 41 years (IQR, [29-49]). The number in each age group was evenly distributed. The median BMI was 22.8 kg/m^2^ (IQR, [20.85-24.45]). The incidence rate of adverse reactions was 28.8%. All of the adverse reactions were minor in severity and resolved within 72 hours after vaccine injection. Among them, pain at the injection site was the most frequently reported local symptom, which was reported in 2 subjects (3.85%), while cough and fatigue were the most frequently reported systemic symptoms, with occurrence rates of 5.77% and 5.77%, respectively.

**Table 1:** Clinical Characteristics of COVID-19 Vaccine Recipients in the Study.

Characteristics	Vaccine recipients (n = 52)
Age, median (IQR), years	41 (29-49)
Sex, n (%)	
Male	28 (53.85%)
Female	24 (46.15%)
BMI, median (IQR), kg/m^2^	22.8 (20.85-24.45)
Solicited Injection-site AE, n (%)	
Numb	1 (1.92%)
Pain	2 (3.85%)
Itch	1 (1.92%)
Solicited systemic AE, n (%)	
Headache	2 (3.85%)
Dry throat	1 (1.92%)
Sore throat	2 (3.85%)
Cough	3 (5.77%)
Fatigue	3 (5.77%)
Comorbidity, n (%)	
Liver disease	8 (15.38%)
Gout	4 (7.69%)
Thyroid disease	9 (17.31%)
Kidney disease	2 (3.85%)
Hyperlipidemia	1 (1.92%)

### Dynamics of Antibody Response to Vaccination

**Figure 2:**
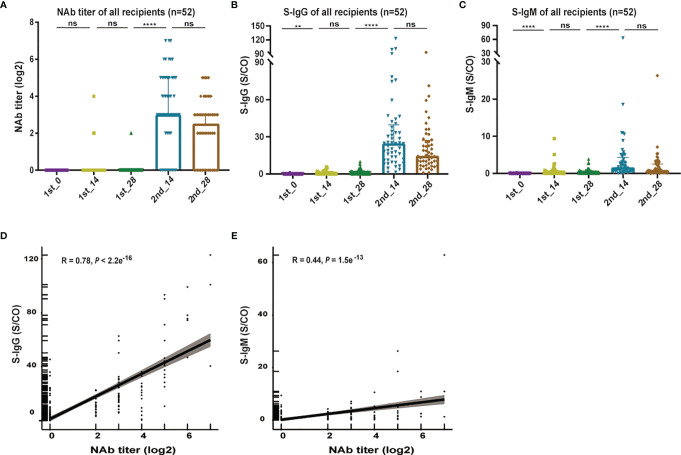
Overall distribution of antibody levels in serum samples at different timepoints after vaccination. **(A–C)**, Overall distribution of anti-SARS-CoV-2 Nab titers **(A)**, S-IgG levels **(B)** and S-IgM levels **(C)** at different timepoints after vaccination. **(D)** Correlation between the NAb titer and S-IgG levels. **(E)** Correlation between the NAb titer and S-IgM levels. A nonparametric Kruskal-Wallis rank-sum method was employed to compare differences in multiple groups. Correlations were analyzed by Pearson correlation coefficients. Statistical significance of the difference between groups is denoted as ** for *P* < 0.01, and **** for *P* < 0.0001. NAb, anti-SARS-CoV-2 neutralizing antibody; S-IgG, SARS-CoV-2 spike-specific immunoglobulin G; S-IgM, SARS-CoV-2 spike-specific immunoglobulin M; ns, not significant.

Serum samples at different timepoints after vaccination showed differences in the overall distribution of antibody levels. As depicted in **Figure 2A**, NAb titers were relatively low or even negative after the first dose; after the second dose, NAb titers were significantly elevated (*P* < 0.0001) at day 14, along with a maximum titer value of 1:128. Despite a decline observed at day 28, the values did not differ significantly between day 14 and day 28 (*P* = 0.5861).

We also evaluated the kinetics of the recipient’s S-IgG and S-IgM development (**Figures 2B, C** and **S1**). The positive rate of S-IgG reached 100% approximately 14 days after the second dose, while the positive rate of S-IgM reached a peak of 69.2% approximately 14 days after the second dose. It shows that the distribution of the S-IgG and S-IgM levels during vaccination were similar to the NAb titer, which were at a low level after the first administration, peaked at day 14 after the second dose and then decreased. Furthermore, correlation analysis showed a good correlation between the S-IgG and NAb titers (R = 0.78, *P* < 2.2 e^-16^, **Figure 2D**), whereas the level of S-IgM (R = 0.44, *P* = 1.5e^-13^, **Figure 2E**) showed a moderate correlation with the NAb titer.

### The Role of Antibody Response and Its Influencing Factors

**Figure 3:**
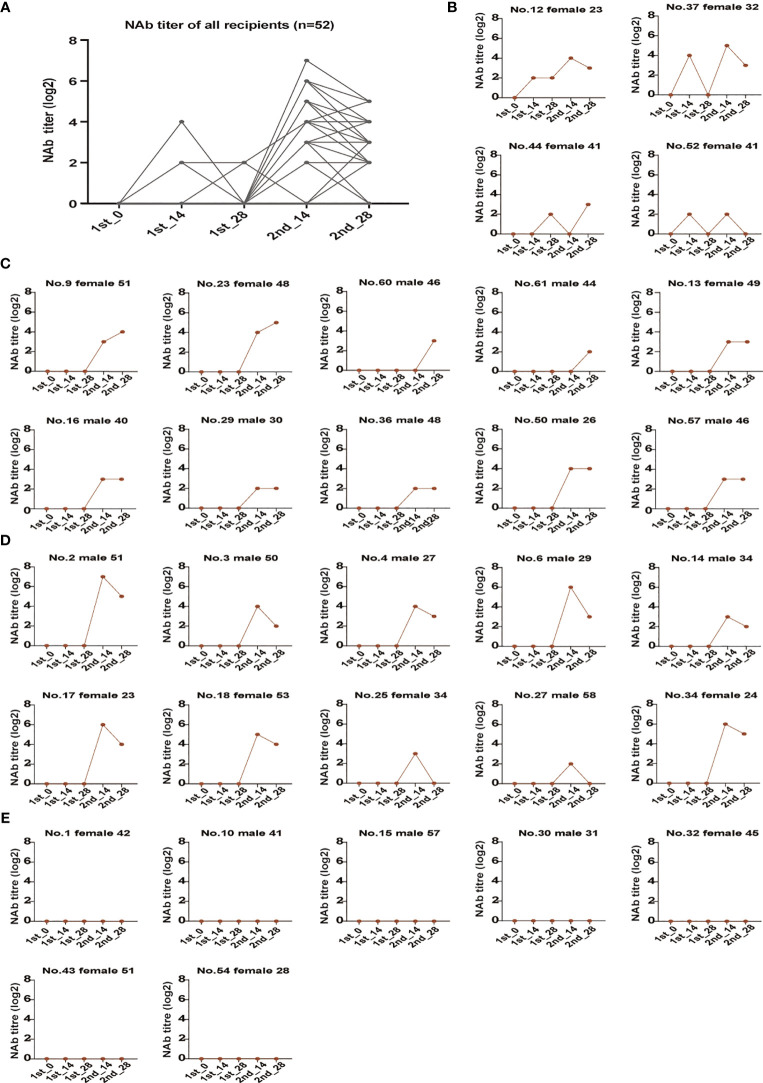
The longitudinal dynamics of the anti-SARS-CoV-2 neutralizing antibody response during the vaccination course. **(A)** Dynamic changes in anti-SARS-CoV-2 NAbs of all recipients (n=52). **(B)** Recipients with an M-shaped dynamic curve of NAb titer levels (n=4). **(C)** Recipients with a slide-shaped dynamic curve of NAb titer levels (n=10). **(D)** Partial recipients with a bell-shaped dynamic curve of NAb titer levels (n=31). **(E)** Recipients with a straight line shape dynamic curve of NAb titer levels (n=7). NAb, anti-SARS-CoV-2 neutralizing antibody.

To explore the role of NAb production after vaccine administration, we analyzed the kinetics of SARS-CoV-2-specific NAb development in each participant during the vaccination course (**Figures 3A** and **S2**). **Figures 3B-E** and **S2** illustrates that the kinetics change of the participants is variable and can be divided into four categories. Four recipients (7.7%) presented an M shape dynamic curve, which developed an NAb titer equal to or slightly above 1:4 before the second dose, followed by a decline (No. 37, No. 44 and No. 52) or stay (No. 12), and then reached the highest value after the next timepoint and dropped mildly again or stay thereafter (**Figure 3B**). The majority of recipients’ NAb titers were negative after the first dose, and started to increase after the second dose (**Figures 3C, D** and **S2**). **Figure 3C** illustrates a dynamic curve with a slide-shaped, revealing a kinetic type (10/52, 19.2%) in which NAb titers further increased or reached a plateau at the timepoint of the last follow-up (2nd-28). However, more recipients (31/52, 59.6%) presented a bell-shaped curve, in which NAb titers mounted a peak after the second dose and then decreased mildly thereafter (**Figures 3D** and **S2**). Notably, seven recipients (13.5%) were found to remain negative at all follow-up timepoints, and the dynamic curve appeared as a straight line (**Figure 3E**).

**Figure 4:**
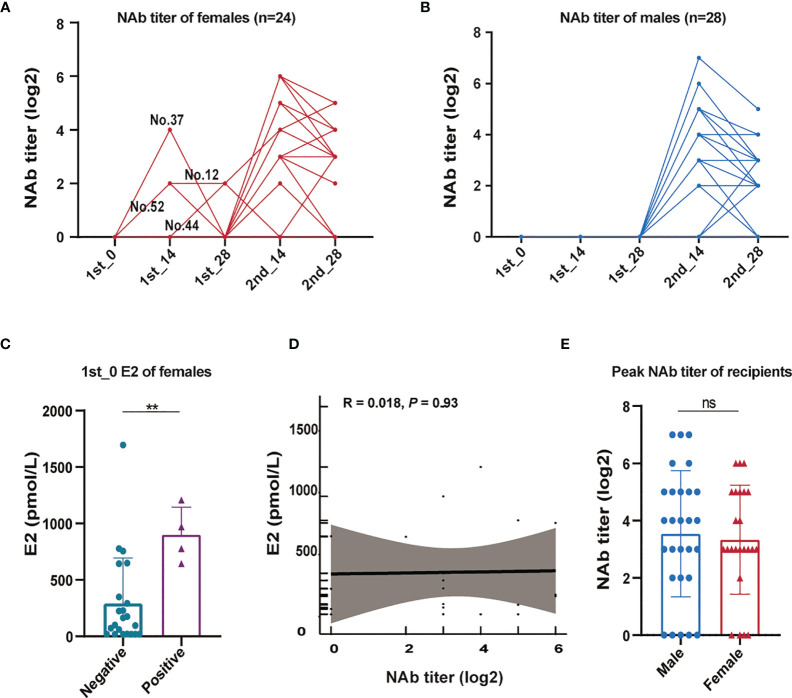
The relationship between sex and NAbs expression. **(A)** Dynamic changes in anti-SARS-CoV-2 neutralizing antibodies in females (n=24). **(B)** Dynamic changes in anti-SARS-CoV-2 neutralizing antibodies in males (n=28). **(C)** Comparison of prevaccine E2 levels between females with or without seroconversion after the first dose. **(D)** Correlation between the peak NAb titer and prevaccine E2 levels. **(E)** Comparison of peak NAb titers between males and females. A nonparametric Mann-Whitney U test was applied to analyze differences between the two groups. Correlations were analyzed by Pearson correlation coefficients. Statistical significance of the difference between groups is denoted as ** for *P* < 0.01. NAb, anti-SARS-CoV-2 neutralizing antibody; E2, estradiol.

Interestingly, we found that vaccinated individuals who presented an M shape curve were all females (**Figure 4A**). None of the male vaccinated individuals developed NAb-positive results before the second dose (**Figure 4B**), indicating that some females may show NAbs earlier than males after vaccination. We sought to determine whether the appearance of NAb responses was related to hormonal mediators and measured the E2 levels prior to vaccination in all females. In comparison, females who were NAb positive after the first dose had significantly higher prevaccine E2 (> 640 pmol/L) than NAb-negative females, indicating that serum E2 may be related to the earlier emergence of NAb (**Figure 4C**). Although the vaccinated females with higher E2 respond to vaccination earlier, there is no significant correlation observed between E2 and peak NAb titer (**Figure 4D**), and males are likely to yield similar peak intensity to that of females (**Figure 4E**).

**Figure 5:**
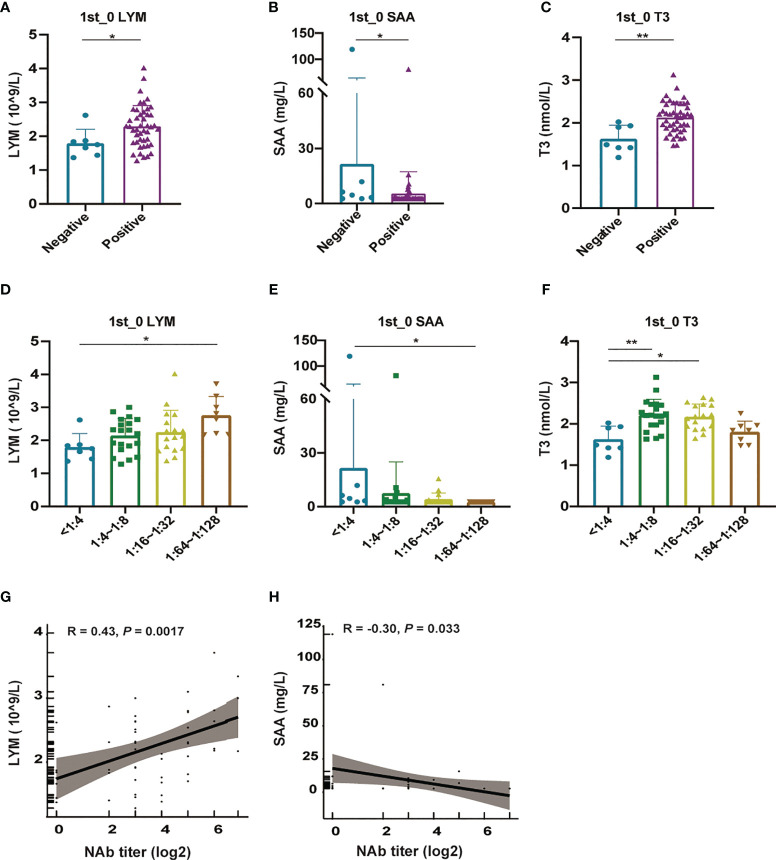
The relationship between the levels of blood parameters and NAb titer levels. **(A-C)**, Comparison of prevaccine lymphocyte counts **(A)**, SAA **(B)**, T3 **(C)** between negative and positive recipients. **(D–F)**, Comparison of prevaccine lymphocyte counts **(D)**, SAA **(E)**, T3 **(F)** between recipients with different peak titer levels. **(G)** Correlation between the peak NAb titer and lymphocyte count levels. **(H)** Correlation between the peak NAb titer and the SAA levels. A nonparametric Kruskal-Wallis rank-sum method was employed to compare differences in multiple groups, and a nonparametric Mann-Whitney U test was applied to analyze differences between the two groups. Correlations were analyzed by Pearson correlation coefficients. Statistical significance of the difference between groups is denoted as * for *P* < 0.05, ** for *P* < 0.01. LYM, lymphocyte counts; SAA, serum amyloid A; T3, triiodothyronine; NAb, anti-SARS-CoV-2 neutralizing antibody.

Despite the overall adequate seroconversion, a straight dynamic curve elicited seven recipients, including 3 males and 4 females, who had no NAb conversions from negative to positive results. To rule out the factors that could have affected NAb seropositivity, we classified the 52 participants into two groups (with seroconversion, n = 43 and without seroconversion, n = 7) and compared the levels of baseline routine blood laboratory parameters between the two groups. Notably, among them, significantly low absolute lymphocyte counts, high levels of serum SAA and low T3 before vaccination were observed in participants without seroconversion (**Figures 5A-C**). As a complementary approach, we further grouped serum into four categories (peak NAb titers: < 1:4; 1:4-1:8; 1:16-1:32; 1:64-1:128) and assessed whether median levels of the above parameters differed across groups. Higher levels of absolute lymphocyte count accompanied by lower serum SAA were also found with higher peak NAb titers (**Figures 5D, E**). The highest levels of T3 were observed in the 1:4-1:8 group (**Figure 5F**). However, no significant difference between NAb titers and other biochemical routine indexes (blood cell counts and differentials, ALT, AST, GGT, CHE, ALP, TBA, LDH, and CRP) or thyroid function markers except T3 was observed (**Figure S3**). We next performed the correlation analyses. Absolute lymphocyte counts in positive relation with peak postvaccination NAb titers are presented in **Figure 5G** (R = 0.43, *P* = 0.0017), whereas a negative correlation between SAA and peak NAb titer is presented in **Figure 5H** (R = -0.30, *P* = 0.033).

**Figure 6:**
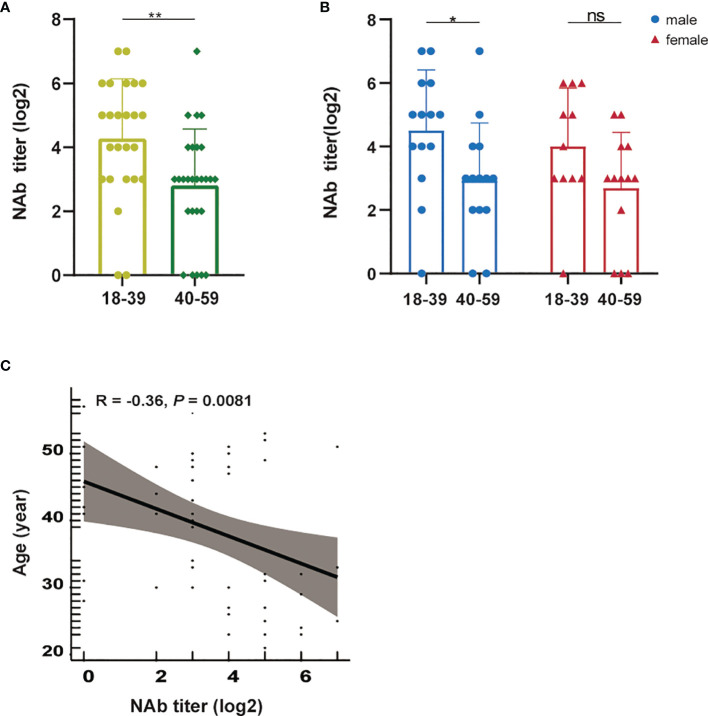
The relationship between age and NAb titer levels. **(A)** Comparison of peak NAb titer levels between 18-39-year-old and 40-59-year-old recipients. **(B)** Comparison of peak NAb titer levels between the 18-39-year-old and 40-59-year-old male and female subgroups. **(C)** Correlation between the peak NAb titer and the age of recipients. A nonparametric Mann-Whitney U test was applied to analyze differences between the two groups. Correlations were analyzed by Pearson correlation coefficients. Statistical significance of the difference between groups is denoted as * for *P* < 0.05, ** for *P* < 0.01. NAb, anti-SARS-CoV-2 neutralizing antibody; ns, not significant.

Considering the important role of age in the immune response, we also explored the relationship between age and NAb titers. We first divided the participants into two groups according to age: 18-39 years (n = 25) and 40-59 years (n = 27) and found that the NAb titers in younger (18-39) vaccine recipients were significantly higher than those in older (40-59, *P* = 0.0026) recipients (**Figure 6A**). A similar trend was observed in the male and female subgroups, although the trend was not significant in the female subgroup (**Figure 6B**). A significant positive correlation was also observed between age and the peak NAb titer (R = -0.36, *P* = 0.0081, **Figure 6C**), confirming the important role of age in the generation of NAbs. A similar development trend was observed in S-IgG and S-IgM (**Figure S1**).

We also measured the participants’ hematologic markers at each timepoint. Except for slight fluctuations in monocyte count and platelet count, there was no significant change in other parameters (**Figure S4**). In addition, the peak NAb titers were negatively correlated with BMI. There was no significant difference in peak NAb titer between recipients with and without comorbidities. No marked difference in peak NAb titer was observed between people vaccinated in the morning (n = 32) and in the afternoon (n = 18). Staying up late (n = 18) or not (n = 32) before vaccination did not affect the highest intensity of NAb (**Figure S5**).

## Discussion

Notably, our results show that the 2 doses of BBIBP-CorV vaccine were immunogenic in healthy adults aged 18-59 years, with seroconversion rates over 90%. We also assessed the dynamics of NAb titers 2 months following vaccination in this well-characterized prospective longitudinal cohort of individuals. The NAb kinetics curves of the participants were classified into four characteristic types, and the related prevaccine factors were preliminarily explored. To our knowledge, our study is the first to elicit different dynamic changes in the NAb with a relatively long duration of follow-up.

NAbs play an important role in virus clearance and have been considered a key immune correlate for protection or treatment against viral diseases (Addetia et al., 2020; Guo et al., 2021). By comparing NAb titers over time with individual follow-up data, we found that the efficacy profile of vaccination might be informed by measurements of NAbs, enabling estimation of protection development. Despite individual differences in antibody level changes, seroconversion was achieved in 90.7% of the subjects, consistent with previous reports (Walsh et al., 2020; Zhang et al., 2020; Pan et al., 2021a). In addition, all the adverse reactions reported here were mild. The safety assessment in our study is comparable to that observed in the BBIBP-CorV phase 1/2/3 trial (Al Kaabi et al., 2021; Isakova-Sivak and Rudenko, 2021) and to other inactivated vaccines, such as CoronaVac (Zhang et al., 2020).

The majority of the participants (78.8%) had a bell-shaped or slide-shaped kinetic curve, which has two properties, 1) the NAb titers were undetectable until the second dose, and 2) the NAb titers peaked at 14 days after the second dose. The inability to detect NAbs after the first dose indicated the need for booster administration, which is consistent with previous studies (Walsh et al., 2020; Zhang et al., 2020). The first dose activates the immune system to produce a partial immune response, and the second dose produces a satisfactory protective immune response (Xia et al., 2021). Regarding the highest titers, the results obtained from phase 1/2 clinical trials of three inactivated SARS-CoV-2 vaccines, namely, BBIBP-CorV, CoronaVac, and KCONVAC, also demonstrated that a strong antibody response was induced at day 14 after the second inoculation, and no significant difference was found at day 28 after the second inoculation (Zhang et al., 2020; Isakova-Sivak and Rudenko, 2021; Pan et al., 2021a). Conversely, a significant reduction in the NAb titer was observed at 14 days after the second dose from the titer at 7 days after the second dose in Walsh’s study, in which the highest NAb titers were measured in samples obtained 7 days after the second dose of RNA-based vaccines (Walsh et al., 2020). However, we should be cautious because most of the studies focus on the general distribution at different timepoints rather than kinetic changes of individuals.

Notably, the NAb titers of 15.4% of participants waned after the initial peak, with negative results obtained at the end of follow-up. This phenomenon was also observed in the studies illustrating individual NAb persistence after natural infections (Beaudoin-Bussières et al., 2020; Seow et al., 2020a; Seow et al., 2020b; Yao et al., 2020). In those studies, Crawford and colleagues concluded that SARS-CoV-2 NAb titers declined approximately four-fold from ~30 to > 90 days after natural infection (Crawford et al., 2020). Huang et al. suggested that approximately 80% of asymptomatic infected people had reduced levels of NAbs within 2 months after infection (Long et al., 2020). Although NAb titer decline with time or even undetectable, we speculate that the protective efficacy with regard to avoiding contracting COVID-19 would not disappear for the following reasons. First, NAbs against SARS-CoV-2 are considered a good indicator of protective immunity but they are not exactly identical to protection. Second, considering that there is currently no widely recognized cut-off value, negative or weak positive results for NAb do not necessarily mean there is no protective power. Third, several studies have shown that antibody responses induced by natural infections might significantly wane (Callow et al., 1990; Amanna et al., 2007; Cao et al., 2007; Wang et al., 2011; Choe et al., 2017; Seow et al., 2020a), such as for SARS-CoV-2, SARS-CoV and MERS-CoV. Nevertheless, the reinfection of these patients has rarely been reported, which indicates that immunological memory (including long-lived plasma cells and memory B cells) might play an important role in preventing reinfection (Cohen et al., 2021; Rodda et al., 2021). In addition, some studies have shown that 4 or more months after the vaccination completion, the antibody titer had decreased substantially, even below the seropositive threshold (Fan et al., 2021; Pan et al., 2021b; Tretyn et al., 2021; Yue et al., 2021). Although Thomas et al. shown a gradually declining trend in vaccine efficacy, spike-specific memory B cells of vaccinators persisted 6 months after vaccination, playing a crucial role in fighting against SARS-CoV-2 infection (Ciabattini et al., 2021; Thomas et al., 2021). After a third booster dose of SARS-CoV-2 vaccine, immune memory can be quickly awakened. Yue et al. demonstrated that the antibody response recovered after seven days, while Pan et al. had shown a 3-5 folds increase in neutralizing antibody titers after the third dose, indicating an anamnestic response (Pan et al., 2021b; Yue et al., 2021). Booster shots to extend the protection of COVID-19 vaccines may be necessary for many people.

Males and females had similar antibody levels in general; nevertheless, remarkably, the antibody response was induced within a shorter time period in some females, whose dynamic curve presented an M shape. These results indicate that a single dose of the vaccine may provide some protection in these women, which is meaningful with regard to certain COVID-19 emergencies. The reason for this phenomenon may be related to differences in hormone levels and is supported by the demonstration that females with higher E2 generated NAb earlier in our study. As reported, E2 enhances both humoral and cell-mediated immune responses (Klein and Flanagan, 2016; Fathi et al., 2020; Park, 2020). There is *in vitro* evidence that high E2 concentrations augment humoral immunity by increasing the number of antibody-secreting cells (Klein et al., 2001). E2 also enhances TH2­type responses and the expansion of Treg cell populations, thus affecting cell immune responses (Klein et al., 2015). Another interesting sex-related phenomenon is that the highest peak titer of NAbs (1:128) was only detected in three male vaccine recipients. However, these phenomena were observed in a small number of people. There were enough participants in the study to strongly demonstrate the relationship between NAb production and sex. Further investigations with larger cohort numbers are required.

It is also noteworthy that 7 subjects presented a straight dynamic shape, that is, NAb positivity after vaccination did not develop. We preliminarily analyzed the factors affecting the production and magnitude of NAb antibodies and found that prevaccine status was likely to play a role. Vaccinated individuals who had higher absolute lymphocyte counts and lower SAA prior to vaccination showed better immune efficacy and elicited higher NAb titers, which may predict a protective response to vaccination (Addetia et al., 2020; Guo et al., 2021). Lymphocytes play a pivotal role in adaptive immunity and are the core component of the immune system. Previous studies demonstrated its impact on vaccination response. Some demonstrated a significant difference in the absolute lymphocyte counts prior to vaccination between patients with vs. without a protective vaccination response (Mavinkurve-Groothuis et al., 2013). Anat Achiron *et al.* reported more direct evidence on the relationship between lymphocyte counts and SARS-CoV-2 vaccination (Achiron et al., 2021). They found that failure to develop SARS-COV-2 antibodies was likely to occur in MS patients who had very low lymphocyte counts. These results indicate that people who are suffering from lymphocytopenia or who are undergoing treatment that appears to reduce lymphocytes should delay vaccination and monitor their absolute lymphocyte counts prior to vaccination. SAA is a key acute phase protein secreted by the liver during the acute phase response following infection or injury. The higher levels of serum SAA in vaccinated individuals without seroconversion than in those with seroconversion were of considerable interest, suggesting that people postpone vaccination if they have recently experienced infection or injury. The result was aligned with the report that SAA is an immunosuppressive factor and suppresses antibody formation and responses (Meretoja et al., 1976). The possible mechanism is that elevated SAA levels are associated with a relative excess of suppressor T lymphocytes (Benson et al., 1975). These findings suggest that an awareness of the link between the kinetics of NAb development and prevaccine blood parameters is needed when developing vaccination strategies.

When comparing antibody levels between age groups, it should be noted that age is another factor affecting NAb titer, and NAb titers decreased significantly with age, similar to the results of previous studies (Walsh et al., 2020; Zhang et al., 2020; Pan et al., 2021a). These findings indicate that an escalated dosage or additional dose of vaccine might be needed in elderly individuals. These results are in accordance with epidemiological trends observed in COVID-19 patients, whereby those with moderate or severe symptoms tended to be elderly individuals (Wu and Mcgoogan, 2020).

The results for S-IgG were comparable to those for Nab, which further confirmed our findings. Although our results showed that NAb titer and S-IgG correlate well, they have their own pros and cons in clinical application. cVNT is the laboratory gold standard for NAb detection; however, this method is limited by the source of live viruses, days to obtain results and the need to operate in high-level biosafety laboratories (Abe et al., 2020; Mendoza et al., 2020). S-IgG and S-IgM test kits are commercially available and can be rapidly performed in most research or clinical laboratories without the need to use live biological materials and biosafety containment; however, they are not capable of measuring specific Nabs (Petherick, 2020; Tan et al., 2020; Tang Y.-W. et al., 2020).

Several limitations of this study should be noted. First, we assessed only serum antibody responses, and further evaluation focusing on memory B cells and cellular immunity is ongoing. Second, the individual specificity of changes in antibody levels needs to be further explored in studies with more subjects and in appropriate designs. Third, we did not compare NAb titers induced by vaccination and convalescent COVID-19 patients in parallel.

In conclusion, our study suggests that vaccination against SARS-CoV-2 can trigger an immune response in the majority of vaccination recipients aged 18 to 59 years. Two doses of the vaccine are necessary to provide sufficient protection. We also gained insight into the dynamic changes in NAb titers and preliminarily explored the prevaccine factors related to the kinetic subclasses, providing a reference for vaccination strategies.

## Data Availability Statement

The raw data supporting the conclusions of this article will be made available by the authors, without undue reservation.

## Author Contributions

JX, WLiu, and DW conceived and designed the experiments. CK, YH, DLiu, LL, YLiu, XT, XZ, WLi, WW, and QK coordinated the projects. JZ, DLia, WH, RY, JH, JL, and YLi collected samples and performed the experiments. JZ, SX, JX, and WLiu. performed the data analysis. SX and JZ wrote the manuscript. All authors contributed to the article and approved the submitted version.

## Funding

This study was supported by the Science and Technology Innovation Project of Foshan Municipality (2020001000431) and the National Key Research and Development Program (2021YFC0863300).

## Conflict of Interest

Author XZ and WLi were employed by the company Autobio Diagnostics Co. Ltd.

The remaining authors declare that the research was conducted in the absence of any commercial or financial relationships that could be construed as a potential conflict of interest.

## Publisher’s Note

All claims expressed in this article are solely those of the authors and do not necessarily represent those of their affiliated organizations, or those of the publisher, the editors and the reviewers. Any product that may be evaluated in this article, or claim that may be made by its manufacturer, is not guaranteed or endorsed by the publisher.

## Abbreviations

COVID-19, coronavirus disease 2019; SARS-CoV-2, severe acute respiratory syndrome coronavirus 2; NAbs, anti-SARS-CoV-2 neutralizing antibodies; S-IgG, SARS-CoV-2 spike-specific immunoglobulin G; S-IgM, SARS-CoV-2 spike-specific immunoglobulin M; BMI, body mass index; ALT, alanine transaminase; AST, aspartate aminotransferase; GGT, gamma-glutamyl transpeptidase; CHE, cholinesterase; LDH, lactate dehydrogenase; TBA, total bile acid; ALP, alkaline phosphatase; SAA, serum amyloid A; CRP, C-reactive protein; E2, estradiol; FT3, free triiodothyronine; FT4, free thyroxine; T3, triiodothyronine; T4, thyroxine; TSH, thyroid-stimulating hormone; cVNT, conventional virus neutralization test; CDC, Centers for Disease Control and Prevention; FBS, fetal bovine serum; DMEM, Dulbecco’s modified Eagle’s medium; TCID 50, 50% tissue culture infectious dose; CPE, cytopathic effects; IQR, interquartile range; ACE2, angiotensin-converting enzyme 2; LYM, lymphocyte count; WBC, white blood cell count; MON, monocyte count; NEU, neutrophil count; NLR, neutrophil-lymphocyte ratio; EOS, eosinophil count; BAS, basophil count; RBC, red blood cell count; PLT, platelet count.
