# Supplementary material for: Differential Antibody Response to Inactivated COVID-19 Vaccines in Healthy Subjects
Source: Front Cell Infect Microbiol. 2021 Dec 16;11:791660. doi: 10.3389/fcimb.2021.791660 (PMC8716725; doi:10.3389/fcimb.2021.791660)
Supplement: Supplementary file 1 [file DataSheet_1.docx]

**Supplementary figures**


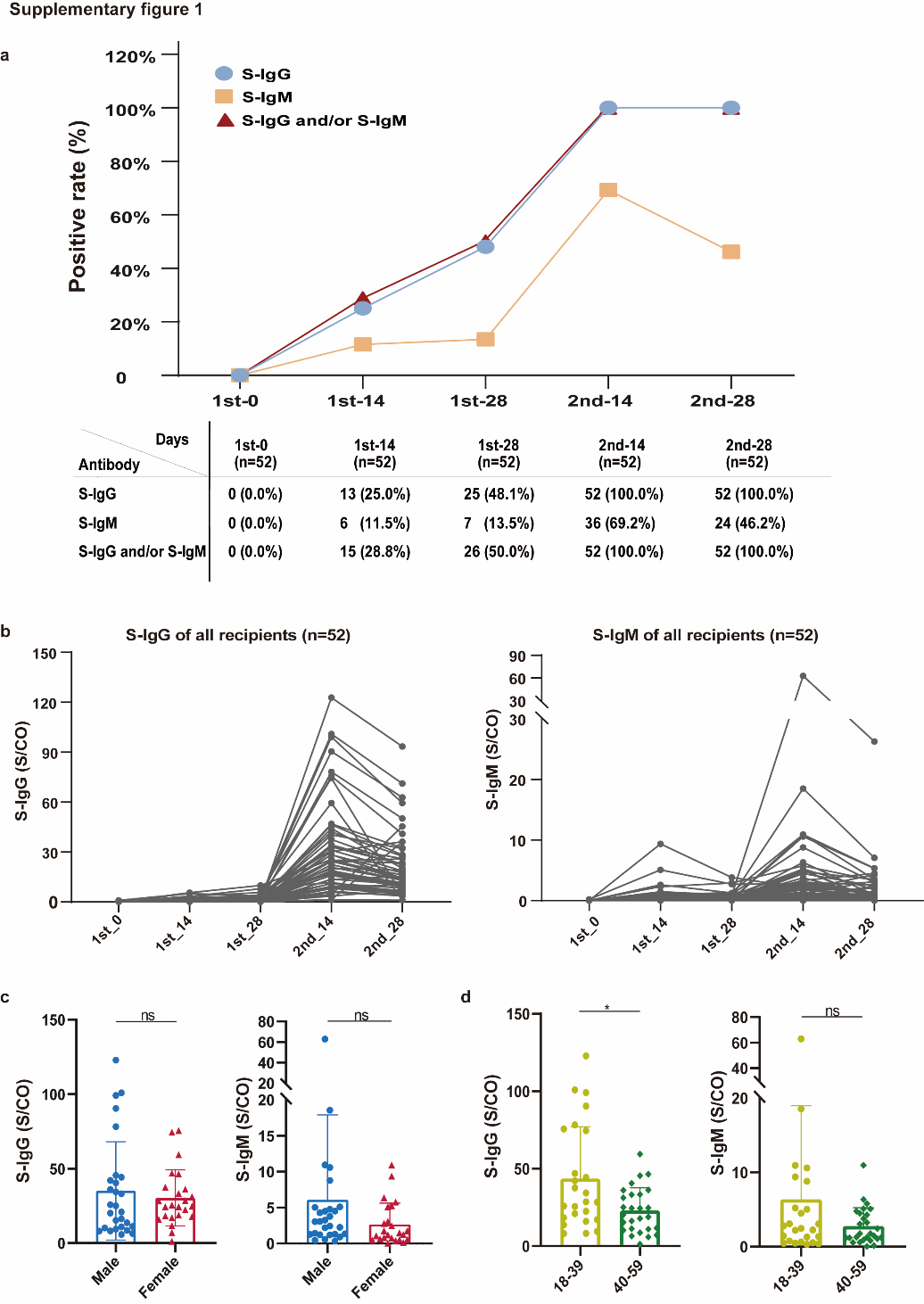


**S1 Fig. S-IgG and S-IgM responses in recipients after vaccination.** a, Positive rates of S-IgG and S-IgM. b, Dynamic changes in S-IgG and S-IgM levels of all recipients. c, Comparison of S-IgG and S-IgM levels between males and females. d, Comparison of S-IgG and S-IgM levels between the 18-39-year-old and 40-59-year-old recipients. A nonparametric Mann-Whitney U test was applied to analyze differences between the two groups. Statistical significance of the difference between groups is denoted as * for P < 0.05. S-IgG, SARS-CoV-2 spike-specific immunoglobulin G; S-IgM, SARS-CoV-2 spike-specific immunoglobulin M.


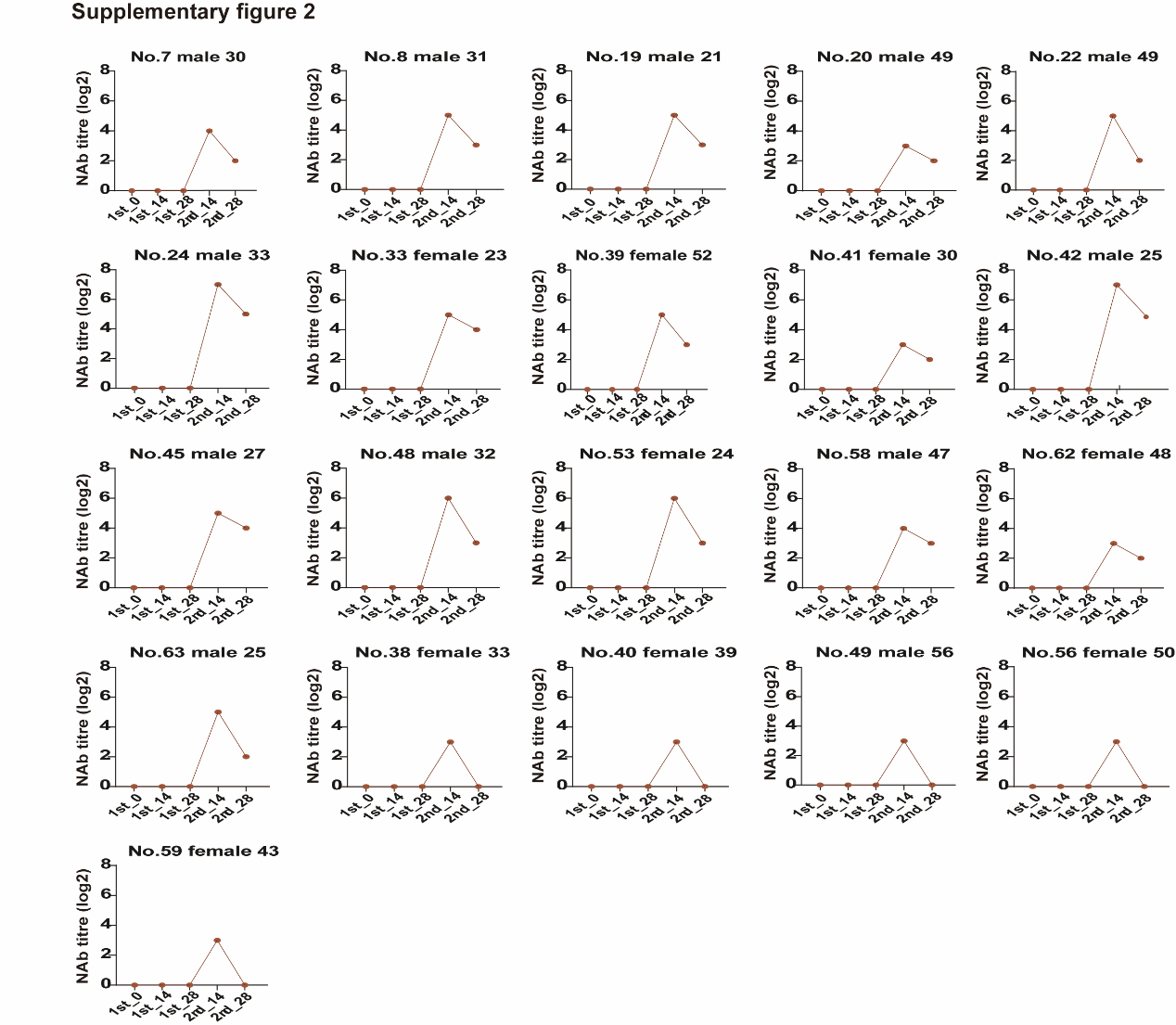


**S2 Fig. Partial recipients with a bell-shaped dynamic curve of NAb titer levels** (supplement to figure 3d). NAb, anti-SARS-CoV-2 neutralizing antibody.


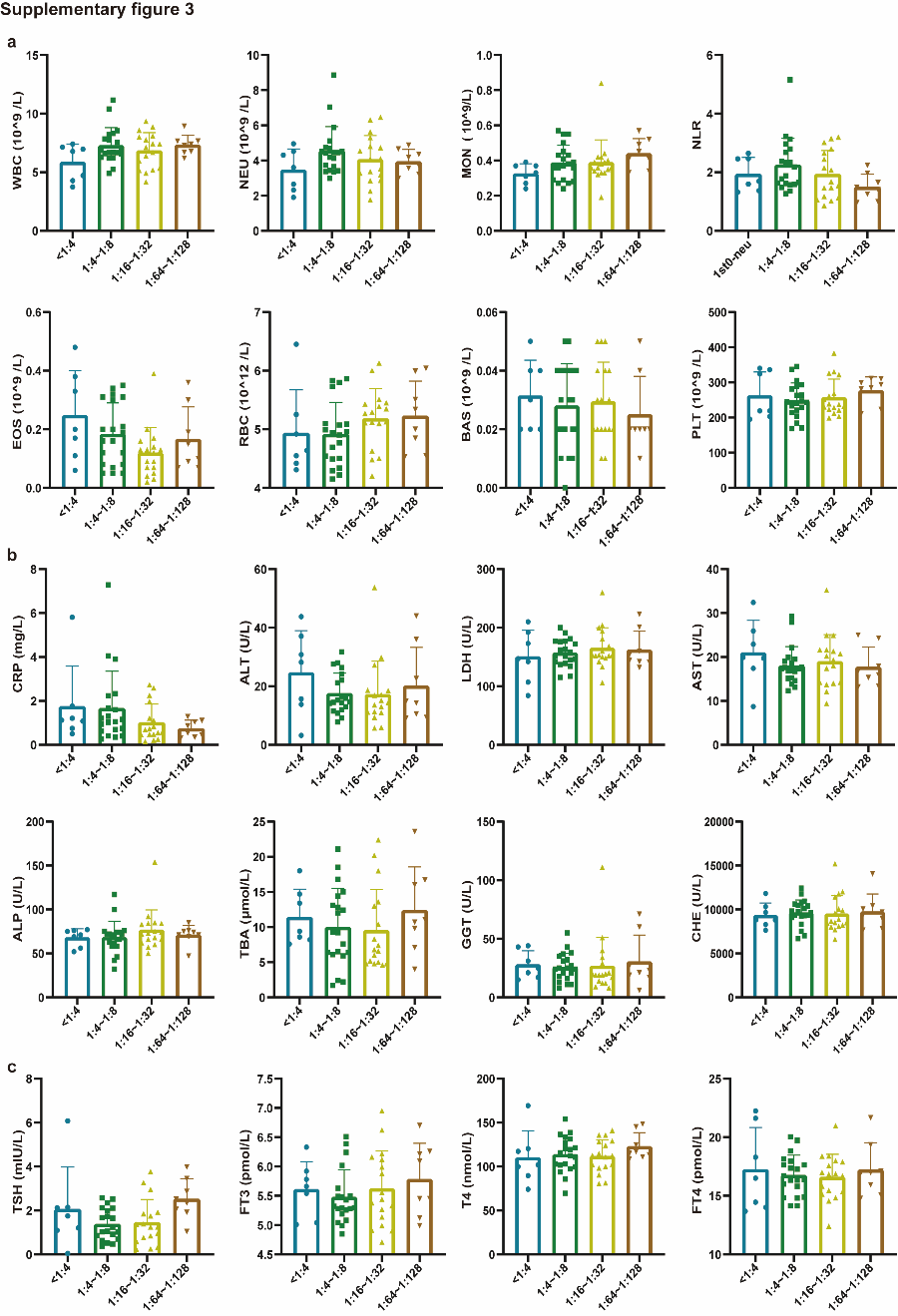


**S3 fig. The routine blood parameter of recipients with different peak NAb titer levels.** a, Prevaccine blood cell counts were analyzed and compared between the groups. b, Prevaccine CRP and liver function indexe were analyzed and compared between the groups. c, Prevaccine thyroid-related hormones were analyzed and compared between the groups. A nonparametric Kruskal-Wallis rank-sum method was employed to compare differences in multiple groups. WBC, white blood count; NEU, neutrophil count; MON, monocyte count; NLR, neutrophil-lymphocyte ratio; EOS, eosinophil count; RBC, red blood cell count; BAS, basophil count; PLT, platelet count; CRP, C-reactive protein; ALT, alanine transaminase; LDH, lactate dehydrogenase; AST, aspartate aminotransferase; ALP, alkaline phosphatase; TBA, total bile acid; GGT, gamma-glutamyl transpeptidase; CHE, cholinesterase. TSH, Thyroid Stimulating Hormone; FT3, Free Triiodothyronine; T4, Thyroxine; FT4, Free Thyroxine.


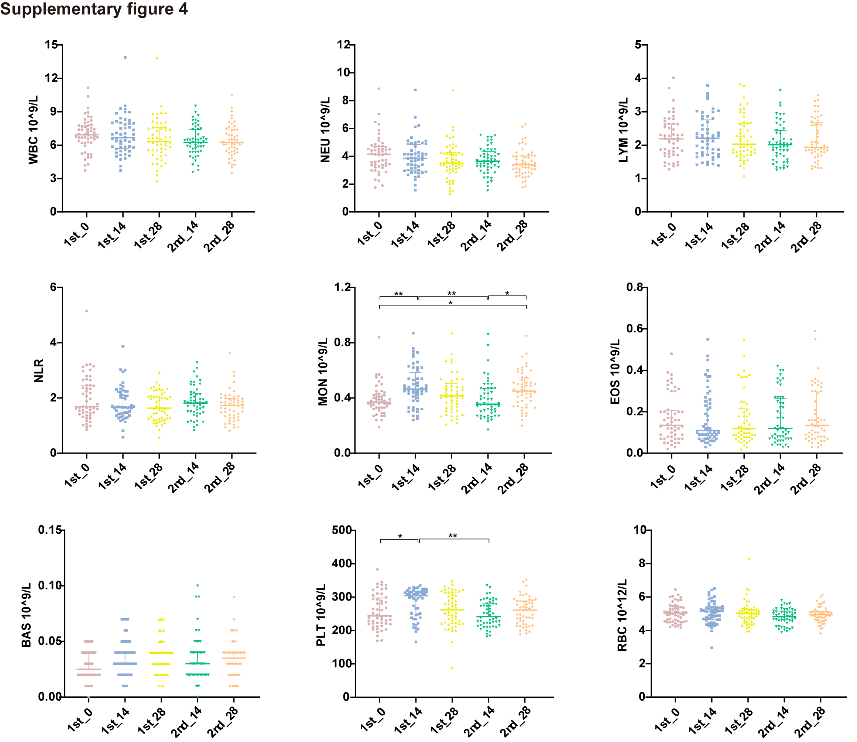


**S4 Fig. Overall distribution of blood cell counts at different timepoints after vaccination**. Overall distribution of WBC, NEU, LYM, NLR, MON, EOS, BAS, PLT and RBC at different timepoints after vaccination. A nonparametric Kruskal-Wallis rank-sum method was employed to compare differences in multiple groups. Statistical significance of the difference between groups is denoted as * for P < 0.05, ** for P < 0.01. WBC, white blood count; NEU, neutrophil count; LYM, lymphocyte; NLR, neutrophil-lymphocyte ratio; MON, monocyte count; EOS, eosinophil count; BAS, basophil count; PLT, platelet count; RBC, red blood cell count.


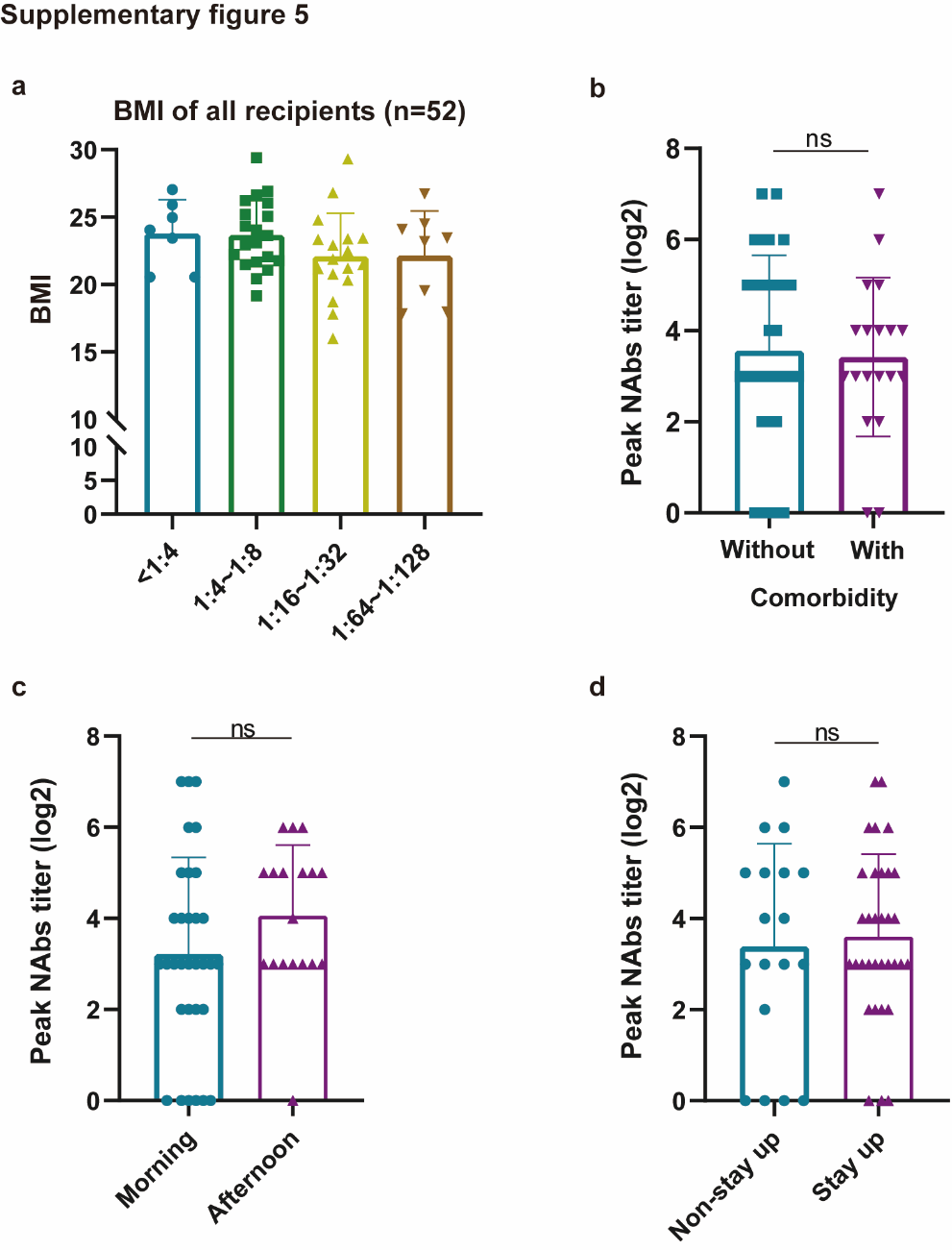


**S5 Fig.** **The relationship between other factors and NAb titer levels**. a, Comparison of BMI between recipients with different peak titer levels. b, Comparison of peak NAb titer levels between recipients with and without comorbidity. c, Comparison of peak NAb titer levels between recipients vaccinated in the morning and in the morning and in the afternoon. d, Comparison of peak NAb titer levels between recipients staying up late and not during vaccination. A nonparametric Kruskal-Wallis rank-sum method was employed to compare differences in multiple groups, and a nonparametric Mann-Whitney U test was applied to analyze differences between the two groups. BMI, body mass index; NAb, anti-SARS-CoV-2 neutralizing antibody.
